# Evolutionarily stable preferences

**DOI:** 10.1098/rstb.2021.0505

**Published:** 2023-05-08

**Authors:** Ingela Alger

**Affiliations:** ^1^ Toulouse School of Economics, University of Toulouse Capitole, 1 esplanade de l'Université, 31080 Toulouse Cedex 06, France; ^2^ CNRS, University of Toulouse Capitole, 1 esplanade de l'Université, 31080 Toulouse Cedex 06, France; ^3^ Institute for Advanced Study in Toulouse, University of Toulouse Capitole, 1 esplanade de l'Université, 31080 Toulouse Cedex 06, France; ^4^ University of Toulouse Capitole, 1 esplanade de l'Université, 31080 Toulouse Cedex 06, France

**Keywords:** altruism, morality, preference evolution, game theory

## Abstract

The 50-year old concept of an evolutionarily stable strategy provided a key tool for theorists to model ultimate drivers of behaviour in social interactions. For decades, economists ignored ultimate drivers and used models in which individuals choose strategies based on their preferences—a proximate mechanism for behaviour—and the distribution of preferences in the population was taken to be fixed and given. This article summarizes some key findings in the literature on evolutionarily stable preferences, which in the past three decades has proposed models that combine the two approaches: individuals inherit their preferences, the preferences determine their strategy choices, which in turn determine evolutionary success. One objective is to highlight complementarities and potential avenues for future collaboration between biologists and economists.

This article is part of the theme issue ‘Half a century of evolutionary games: a synthesis of theory, application and future directions’.

## 1. Introduction

What drives the behaviour of humans in their interactions with others? The premise in evolutionary game theory is that each individual is *programmed* to use a certain strategy. Since the typical life of a human being consists of a large number of different kinds of interactions, nature should thus have equipped us with automatic play of a certain strategy tailored to each one of them, the *ultimate driver* of the strategies played in a population being natural selection [1]. Such a worldview is, however, at odds with the idea that we both *understand* the situations we find ourselves in and *choose* how to act. But if the latter is an accurate description of how strategies are selected, what then guides the strategy choice?

One theory comes from economics, where the overwhelmingly common premise is that each individual has preferences over the available strategies, which simply means that if presented with a pair of strategies, say *A* and *B*, (s)he can tell whether (s)he prefers *A* to *B*, (s)he prefers *B* to *A*, or is indifferent between the two strategies. In an interaction with others, the answer may depend on what strategies the others are expected to play. Rational behaviour requires that a strategy that is preferred over the others be selected by the individual. A Nash equilibrium strategy profile is such that no interactant prefers to alter his/her strategy given the opponents’ strategies. In this approach, the individual’s preferences are the *proximate driver* of his/her behaviour.

When combining these two strands of thought, the question that follows naturally is: if humans choose strategies in accordance with their preferences, which preferences should we expect evolutionary forces to favour, if any? The literature on preference evolution, initiated by Frank [2] and Güth & Yaari [3], provides some answers to this question. This article summarizes some of the key findings of this literature, found mostly in economics journals, and draws some parallels with related contributions by biologists, notably McNamara *et al.* [4], Taylor & Day [5], Akçay *et al.* [6] and Hamilton [7,8].

## 2. Strategy evolution in biology

### (a) Framework and definition of evolutionarily stable strategy

Throughout I adopt the following assumptions, which are in line with the standard evolutionary game theory model [9]. First, I follow John Maynard Smith by defining a '‘strategy’ [as] a behavioural phenotype, i.e. it is a specification of what an individual will do in any situation in which it may find itself’ ([10, p. 10], see also the recent book by McNamara & Leimar [11]); this is also in line with standard vocabulary in non-cooperative game theory (see [12]). Second, I examine a continuum population, in which individuals are randomly matched into pairs to interact, i.e. there is no partner choice. Third, I restrict attention to interactions in which both individuals have access to the same set of strategies, called *X*, and the material consequences of strategy choices are identical for all individuals in the population. Letting *w*(*x*, *y*) denote the (personal) *fitness* of an individual using strategy *x* when the other is using strategy *y*, I will refer to Γ=⟨X,w⟩ as the *fitness game*.^1^ The question at hand is whether some *resident strategy* *x*, present in a share 1−ε of the population, would resist the invasion of some *mutant strategy* *y*, present in a small share ε>0 of the population. An evolutionarily stable strategy (ESS) is then formally defined as follows [9]:

**Definition 2.1.**

Consider a population in which individuals are uniformly randomly matched into pairs to interact according to the fitness game Γ=⟨X,w⟩. A strategy *x* ∈ *X* is **evolutionarily stable (ES)** against strategy *y* ∈ *X*, *y* ≠ *x*, if there exists ${\bar{\varepsilon }}_{y}\in (0,1)$ such that for all $\varepsilon \in (0,{\bar{\varepsilon }}_{y})$:

2.1 $$\begin{array}{r} (1-\varepsilon )\cdot w(x,x)+\varepsilon \cdot w(x,y)>(1-\varepsilon )\cdot w(y,x)+\varepsilon \cdot w(y,y). \end{array}$$

And *x* is an **ESS** if it is ES against all *y* ∈ *X*, *y* ≠ *x*. ■

The population being infinitely large and the interactants being matched in a uniformly random manner, any individual is matched with a resident (who plays *x*) with probability 1−ε and with a mutant (who plays *y*) with probability ε. In (2.1), the left-hand side is thus the average fitness of individuals playing the resident strategy, while the right-hand side is the average fitness of individuals playing the mutant strategy, given the share ε of mutants in the population. In words, then, an ESS is a strategy that, once it has become prevalent in a population, earns a higher average fitness than any rare mutant strategy.

**Remark 2.2.**

The present model disregards a number of features that are present in many rich mathematical models in evolutionary biology, in which: the process by which individuals are matched to interact depends on the life-cycle and the population structure; there are individuals of different classes (e.g. men and women, old and young, etc.); there are explicit computations of the expected number of descendants of a single initial mutant; etc. For a recent such general model, see Lehmann & Rousset [13]. The model in definition 2.1 is closely related to the special case of theirs, in which there is full dispersal, haploidy and weak selection, the latter assumption ensuring that the probability of being matched with a mutant does not depend on the mutant strategy itself. In this case, the invasion fitness (see their eqn (2)) reduces to *w*(*y*, *x*)/*w*(*x*, *x*), and the condition for *x* to be uninvadable (see their eqn (1)) is equivalent to *w*(*y*, *x*) ≤ *w*(*x*, *x*), a condition that below will be seen to be necessary for *x* to be ESS.^2^ This bare-bones model presents the advantage of making it easy to derive insights on preference evolution and understand the challenges that its analysis presents. ■

While much of the literature focuses on one-shot simultaneous-move games with a finite number of strategies, such as the Prisoner’s Dilemma or the Hawk–Dove game, the setting in fact encompasses a large number of other kinds of interactions, for instance those with an infinite number of pure strategies, and/or where the interactants play in a sequential manner. Several such games are described in detail in electronic supplementary material, §S1.^3^ While several general results will be presented, I will often refer to the following two examples.

**Example 2.3.**

(Fitness game $\Gamma_{1}$) This is a (simultaneous-move one-shot) public goods game with strategy set $X=R_{+}$ and fitness function

2.2 $$w(x,y)=(m+ky)x-x^{2}.$$

The first term is the benefit and the second term the cost from contributing *x*. The parameter *m* > 0 measures the baseline marginal benefit from the contribution. The parameter *k* ∈ ( − 1, 1) measures the effect that the other individual’s contribution *y* has on the marginal benefit from the contribution: if *k* ∈ ( − 1, 0), *y* reduces the marginal benefit by *ky*; if *k* ∈ (0, 1) it increases the marginal benefit by *ky*; finally, if *k* = 0 it has no effect on the marginal benefit. ■

Note that the parameter *k*, which determines how the other’s contribution affects the individual’s marginal benefit from contributing, is nothing but the cross-partial derivative of *w*: ∂^2^ *w*(*x*, *y*)/(∂*x*∂*y*) = *k*. The following definition proposes a general classification of fitness games depending on the sign of this cross-partial derivative.^4^

**Definition 2.4.**

In a given fitness game Γ=⟨X,w⟩, the strategies are:

1. strategic substitutes if ∂^2^ *w*(*x*, *y*)/(∂*x*∂*y*) < 0 for all (*x*, *y*) ∈ *X*^2^;
2. strategic complements if ∂^2^ *w*(*x*, *y*)/(∂*x*∂*y*) > 0 for all (*x*, *y*) ∈ *X*^2^;
3. strategically neutral if ∂^2^ *w*(*x*, *y*)/(∂*x*∂*y*) = 0 for all (*x*, *y*) ∈ *X*^2^. ■

Hence, in example 2.3 the strategies are strategic substitutes if *k* ∈ ( − 1, 0), strategically neutral if *k* = 0, and strategic complements if *k* ∈ (0, 1). Instances of interactions involving strategic substitutes are those where individuals compete over the same resources, such as common-pool resource games, or where individuals benefit from free-riding on each other, such as commonly studied public goods games. By contrast, strategic complementarities are present in interactions where it pays to coordinate, i.e. where teamwork is valuable. This leads us to example 2.5.

**Figure 1.**
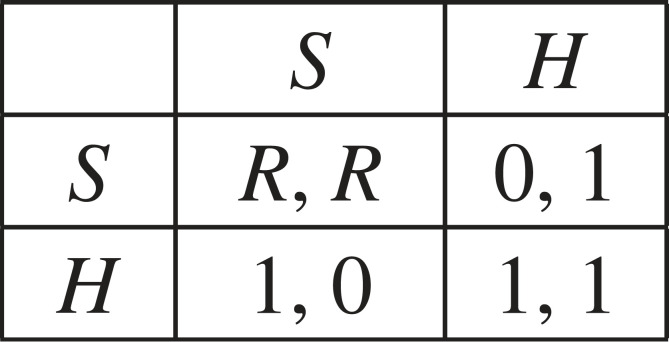
The payoff matrix of the simultaneous-move Stag hunt game (*R* > 1).

**Example 2.5.**

(Fitness game $\Gamma_{2}$) This is the (simultaneous-move one-shot) Stag hunt game [12], in which each individual has two actions—Stag (*S*) and Hare (*H*), and with payoffs as shown in figure 1. This game captures interactions in which *H* (‘going for the Hare’ in the original story) gives a certain payoff of 1 but coordination by both players on *S* (going for the Stag) leads to a higher payoff *R* > 1. A (mixed) strategy is the probability of playing *S*, with *H* being played with the complementary probability. Hence, the strategy set is *X* = [0, 1] and the fitness function is

2.3 w(x,y)=xyR+1−x.

While the strategy labels stem from the original story of two hunters, this fitness game can be used to represent a host of interactions where coordination on one strategy enhances the fitness values of both individuals. ■

### (b) An ‘as if’ interpretation of evolutionarily stable strategy

As a first step towards analysis of preference evolution, it is worth noting that a population in which an ESS is played can be viewed as being populated by individuals who seek to maximize own fitness.

To see this—and also to facilitate description of the analytical challenges that preference evolution sometimes entails—it proves useful to express the difference between the average fitness earned by residents and that earned by mutants as a function of the share of mutants ε, using what is called the *score function* [14]:

2.4 $$\begin{array}{r} S_{x,y}(\varepsilon )=(1-\varepsilon )\cdot [w(x,x)-w(y,x)]+\varepsilon \cdot [w(x,y)-w(y,y)]. \end{array}$$

This function being linear in ε, *S*_*x*,*y*_(0) ≥ 0 is a necessary condition for *x* to be ES against *y*, while *S*_*x*,*y*_(0) > 0 is a sufficient condition.^5^ Moreover, if *S*_*x*,*y*_(0) = 0 then the slope of the score function must be strictly positive for *x* to be ES against *y*. This leads to the following result and also simple test for whether a strategy is ES:

**Result 2.6.**

1. If *w*(*x*, *x*) > *w*(*y*, *x*), then *x* is ES against *y*.
2. If *w*(*x*, *x*) = *w*(*y*, *x*), then *x* is ES against *y* if and only if *w*(*x*, *y*) > *w*(*y*, *y*).
3. If *w*(*x*, *x*) < *w*(*y*, *x*), then *x* is not ES against *y*. ■

As an illustration, in example 2.3 the unique ESS is *x*^ESS^ = *m*/(2 − *k*). This is found by noting that any ESS *x*^ESS^ must solve

2.5 $$x^{\mathrm{ESS}}\in \arg\max_{y\in X}w(y,x^{\mathrm{ESS}}).$$

The first-order condition for such a maximum is

2.6 $${\frac{\partial w(y,x^{\mathrm{ESS}})}{\partial y}}_{|y=x^{\mathrm{ESS}}}={\left(m+kx^{\mathrm{ESS}}-2y\right)}_{|y=x^{\mathrm{ESS}}}=0.$$

This equation has as unique solution *x*^ESS^ = *m*/(2 − *k*). Since the second-order derivative with respect to *y* is strictly negative, it follows that *w*(*x*^ESS^, *x*^ESS^) > *w*(*y*, *x*^ESS^) for any *y* ≠ *x*^ESS^. Result 2.6 then implies that *x*^ESS^ is ES against any *y* ∈ *X*, *y* ≠ *x*^ESS^, and definition 2.1 in turn implies that *x*^ESS^ is an ESS. In example 2.5, there are two ESSs: play *S* with probability 1, and play *H* with probability 1.

Since *x* is ES only if *w*(*x*, *x*) ≥ *w*(*y*, *x*) for all *y* ≠ *x*, an individual in a population where the ESS *x* is played by everyone can be interpreted *as if* (s)he were choosing the strategy that maximizes his/her fitness, given that any individual (s)he interacts with uses strategy *x*. This observation brings us to the main question: what if, instead of equipping us with automatic play of a strategy tailored to each possible interaction, nature has equipped humans with (a) the ability to understand the situations they find themselves in, and (b) some *preferences* that guide their strategy choice in any given interactions? Which preferences should we then expect evolutionary forces to favour, if any?

## 3. Preference evolution

### (a) In economics, preferences guide behaviour

In their analyses of human behaviour, economists typically rely on the premise that in any given situation each individual chooses the option that (s)he prefers among all the options that are feasible for him/her. Choosing an option other than a preferred one is deemed irrational. This simple idea is captured by positing that each individual is able to rank all the feasible options. This ranking is then formalized as a preference ordering, which for any pair of feasible options *A* and *B* tells whether the individual prefers *A* or *B*, or is indifferent between *A* and *B*. It turns out that under certain conditions, such a preference ordering can be fully described by a function that associates a real number with each feasible option: the number associated with option *A* is higher (respectively lower) than that associated with option *B* if and only if the individual prefers *A* over *B* (respectively *B* over *A*), and the same number for both options if the individual is indifferent between them (e.g. [15]). Such a function is called a *utility function* in economics. Here I will instead refer to it as a *preference function*. In any given situation, an individual is expected to choose the option that yields the highest possible value of the function, since this is the option (s)he prefers. Economists do not interpret this utility maximization literally: it is simply a mathematical tool that the researcher uses to describe behaviour that amounts to choosing the preferred item from the *feasible set*.

In the context of a fitness game Γ=⟨X,w⟩, an individual’s feasible set is the set of strategies *X*. The individual with whom (s)he is matched to interact—the opponent—also chooses some strategy in the strategy set *X*. To capture the fact that an individual’s ranking over own strategies may depend on what strategy the opponent is expected to use, a preference function is some function $u\;:\;X^{2}\to R$ that associates a real number with each pair of own and opponent’s strategies. If the individual strictly prefers some strategy profile, say (*x*, *y*), over another, say (*x*′, *y*′), then *u* gives a strictly higher number to the former, while if the individual is indifferent, then *u* gives the same number to both strategy profiles. The question posed at the end of the previous section can now be formally stated as follows: given that personal fitness drives evolutionary success, should we expect evolution to favour the preference function that induces the individual to maximize personal fitness?

**Definition 3.1.**

In any given fitness game Γ=⟨X,w⟩, **a (personal) fitness-maximizer** has a preference function that coincides with the personal fitness function, i.e.

3.1 u(x,y)=w(x,y)for all strategies  x,y∈X.

For any given strategy that a fitness-maximizer expects the opponent to choose, (s)he chooses a strategy that maximizes own fitness, since this is the strategy that (s)he is encoded to prefer. Should we expect evolution to lead humans to be such *fitness-maximizers*? The literature has revealed that information plays a key role in this context. ■

### (b) Interactions under complete information

An example will serve as an introduction. Consider a population in which individuals are matched pairwise to interact according to a common-pool resource game, formalized as fitness game $\Gamma_{1}$ with parameter values *m* = 10 and *k* = −1/2 (see (2.2)). Having in mind situations such as fishing in a common lake, I will use ‘extraction effort’ or simply ‘effort’ to refer to a strategy. The negative value of *k* means that an individual’s effort has a negative impact on the other’s marginal return to effort, the idea being that this effort diminishes the size of the fish population available to the other.

Suppose first that all individuals in the population are fitness-maximizers, i.e. each individual has preferences $u\;:\;X^{2}\to R$ defined in (3.1), namely *u*(*x*, *y*) = (10− *y/2*)*x* − *x*^2^, over own and other’s efforts, *x* and *y*. Suppose further that individuals who are matched to interact can observe each other’s preference function, i.e. they interact under *complete information* [12]. In such an interaction, what pair of strategies will be played? While this question has no simple general answer (e.g. [16]), it is common in economics to apply the *Nash equilibrium concept*.

**Definition 3.2.**

In an interaction between two individuals with preferences $u\;:\;X^{2}\to R$ and $v\;:\;X^{2}\to R$, respectively, a pair of strategies (x*, y*) is a *Nash equilibrium* if neither individual would like to deviate from their strategy, given the other’s strategy. Formally,

3.2 $$\begin{array}{cc} & x^{*}\in \underset{x\in X}{\arg\max}\;u(x,y^{*})\\ \mathrm{and}\; & y^{*}\in \underset{y\in X}{\arg\max}\;v(y,x^{*}). \end{array}\}$$

In the example, in an interaction between two fitness-maximizers, (x*, y*) is a Nash equilibrium if and only if:

3.3 $$\begin{array}{cc} & x^{*}\in \underset{x\in X}{\arg\max}\;(10-y^{*}/2)x-x^{2}\\ \mathrm{and}\; & y^{*}\in \underset{y\in X}{\arg\max}\;(10-x^{*}/2)y-y^{2}. \end{array}\}$$

Differentiability of the preference function together with an unbounded strategy set implies that (x*, y*) must satisfy the following set of the first-order conditions for x* and y* to be maxima (these conditions are also sufficient because of the strict concavity of the preference function):

3.4 $$\begin{array}{cc} & 10-\frac{y^{*}}{2}=2x^{*}\\ \mathrm{and}\; & 10-\frac{x^{*}}{2}=2y^{*}. \end{array}\}$$

It follows that the unique Nash equilibrium strategy profile between two fitness-maximizers is (x*, y*) = (4, 4) (note that this is also the ESS *x*^ESS^ = *m*/(2 − *k*)). Suppose now that another preference function enters this population. For example, suppose that some individuals have the preference function

3.5 $$v(x,y)=w(x,y)-\frac{1}{2}w(y,x).$$

For any given strategy used by the individual, *x*, and any given strategy used by the opponent, *y*, in this function both the individual’s own fitness, *w*(*x*, *y*), and the fitness of the opponent, *w*(*y*, *x*), appear. Since the former enters with a positive sign and the latter with a negative sign, this function means that the individual prefers strategy profiles (*x*, *y*) that give a higher fitness to itself and a lower fitness to the opponent. In economics such an individual is said to have *spiteful preferences* (e.g. [17]). In an interaction between one fitness-maximizer (with preference function *u*) and one spiteful individual (with preference function *v*) a pair of strategies $\left(\hat{x},\hat{y}\right)$ is a Nash equilibrium if and only if:

3.6 $$\begin{array}{rl} & \hat{x}\in \underset{x\in X}{\arg\max}\;\left(10-\frac{\hat{y}}{2}\right)x-x^{2}\\ \mathrm{and}\; & \hat{y}\in \underset{y\in X}{\arg\max}\;\left(10-\frac{\hat{x}}{2}\right)y-y^{2}-\frac{1}{2}\left[\left(10-\frac{y}{2}\right)\hat{x}-{\hat{x}}^{2}\right]. \end{array}\}$$

The following first-order conditions are necessary (and also sufficient because of the strict concavity of the preference functions):

3.7 $$\begin{array}{rl} & 10-\frac{\hat{y}}{2}=2\hat{x}\\ \mathrm{and}\; & 10-\frac{\hat{x}}{2}+\frac{\hat{x}}{4}=2\hat{y}. \end{array}\}$$

It follows that the unique Nash equilibrium strategy profile is $\left(\hat{x},\hat{y})=(120/31,140/31)\approx (3.87,4.52\right)$. Note that $\hat{y}>x^{*}>\hat{x}$. By attaching a negative weight to the other’s fitness, the spiteful individual extracts more from the common-pool resource than a fitness-maximizer; in turn, compared with when (s)he interacts with another fitness-maximizer and makes extraction effort x* = 4, here the fitness-maximizer compensates for the higher extraction effort by his/her opponent by decreasing his/her extraction effort, to $\hat{x}\approx 3.87$, because the marginal benefit from extracting is reduced when the opponent is extracting more. Calling the preference function *u* the resident trait, and the spiteful preference function *v* the mutant trait, and letting ε denote the share of individuals with the mutant trait, the average fitness of the resident fitness-maximizers is

3.8 $$\left(1-\varepsilon )\cdot w(x^{*},x^{*})+\varepsilon \cdot w(\hat{x},\hat{y}\right)$$

while the average fitness of the mutant spiteful individuals is

3.9 $$(1-\varepsilon )\cdot w(\hat{y},\hat{x})+\varepsilon \cdot w(\tilde{x},\tilde{x}),$$

where $\left(\tilde{x},\tilde{x})=(40/9,40/9)\approx (4.44,4.44\right)$ is the unique Nash equilibrium strategy profile in a match between two spiteful individuals. Since $(140\times 110)/31^{2}=w(\hat{y},\hat{x})>w(x^{*},x^{*})=16$, it follows that for ε close enough to zero, the mutants obtain a strictly higher average fitness than the residents. Following the same logic as in standard evolutionary game theory, I conclude that a population of fitness-maximizers would not resist the invasion by mutants with the spiteful preference function *v*.

The conclusion that a population of fitness-maximizers would not resist the invasion by some other preference function, here reached in a simple example, has been shown by Heifetz *et al.* [18] to hold for any fitness game Γ=⟨X,w⟩ such that *w* is a thrice differentiable function and *X* is an open subset of R (see also Ok & Vega-Redondo [19]). They show this general result in a model that encompasses any preference function of the form

3.10 u(x,y)=w(x,y)+B(x,y,τ),

where τ∈E⊆R is the evolving trait and *B* is some thrice differentiable function (the fitness-maximizer is the special case with *B*(*x*, *y*, *τ*) = 0 for all (*x*, *y*) ∈ *X*^2^). ■

This then leads to the question: which preference function, if any, is ES?

**Definition 3.3.**

Consider a population in which individuals are uniformly randomly matched into pairs to interact according to the fitness game Γ=⟨X,w⟩ under complete information about each other’s preferences. Let Θ denote the set of all possible preference functions $u\;:\;X^{2}\to R$; any u∈Θ is such that there exists a unique Nash equilibrium in each matched pair. Then, a preference function u∈Θ is **ES under complete information (ESC)** against preference function v∈Θ if there exists ${\bar{\varepsilon }}_{v}\in (0,1)$ such that for all $\varepsilon \in (0,{\bar{\varepsilon }}_{v})$:

3.11 $$\begin{array}{r} (1-\varepsilon )\cdot w(x^{*},x^{*})+\varepsilon \cdot w(\hat{x},\hat{y})>(1-\varepsilon )\cdot w(\hat{y},\hat{x})+\varepsilon \cdot w(\tilde{x},\tilde{x}), \end{array}$$

where (x*, x*) is the unique Nash equilibrium in an interaction between two residents, $\left(\hat{x},\hat{y}\right)$ is the unique Nash equilibrium in an interaction between a resident and a mutant, and $\left(\tilde{x},\tilde{x}\right)$ is the unique Nash equilibrium in an interaction between two mutants.

The preference function *u* is an **evolutionarily stable preference function under complete information (ESPFC)** if it is ES against all preference functions v∈Θ, *v* ≠ *u*. ■

In words, an ESPFC is a preference function that, once it has become prevalent in a population, cannot be displaced by any other preference function, the criterion being fitness evaluated at Nash equilibrium.^6^ It should be remarked that the definition can be generalized to encompass settings where there exist multiple Nash equilibria; however, most of the literature has restricted attention to settings with a unique Nash equilibrium (an exception is the model of Dekel *et al.* [20], but their analysis is on the other hand restricted to fitness games with finite action sets).

In settings where there exists a unique Nash equilibrium in each matched pair, the score function is well defined:

3.12 $$\begin{array}{r} S_{u,v}(\varepsilon )=(1-\varepsilon )\cdot [w(x^{*},x^{*})-w(\hat{y},\hat{x})]+\varepsilon \cdot [w(\hat{x},\hat{y})-w(\tilde{x},\tilde{x})]. \end{array}$$

Since *S*_*u*,*v*_ is linear in ε, the following result and simple test obtains:

**Result 3.4.**

Let (x*, x*) be the unique Nash equilibrium in an interaction between two residents, $\left(\hat{x},\hat{y}\right)$ the unique Nash equilibrium in an interaction between a resident and a mutant, and $\left(\tilde{x},\tilde{x}\right)$ the unique Nash equilibrium in an interaction between two mutants. Then:

1. If $w(x^{*},x^{*})>w(\hat{y},\hat{x})$, then *u* is ESC against *v*.
2. If $w(x^{*},x^{*})=w(\hat{y},\hat{x})$, then *u* is ESC against *v* if and only if $w(\hat{x},\hat{y})>w(\tilde{x},\tilde{x})$.
3. If $w(x^{*},x^{*})<w(\hat{y},\hat{x})$, then *u* is not ESC against *v*. ■

A fundamental difference with strategy evolution is that the set of potential preference functions, Θ, is *a priori* undetermined. Hence, the researcher must make some assumption. Thus by far most of the analyses of preference evolution under complete information have examined the parametric class of preferences originally proposed by Bester & Güth in their seminal paper [21]. (For analyses of other preference functions, see [22] and [23].) In a model with the fitness function given in (2.2), they examine preference functions of the form

3.13 $$u_{\alpha }(x,y)=w(x,y)+\alpha \cdot w(y,x),$$

where *α* ∈ [0, 1] is the evolving trait. Bolle [24] and Possajennikov [25] generalized the original model by extending the range of possible values of *α* to R. Like in the example studied in detail above (which corresponds to the special case *α* = −1/2), a straightforward interpretation is that an individual with such a preference function attaches some weight, *α*, to the consequences of his/her strategy choice on the opponent's fitness. If *α* > 0, (s)he is willing to reduce own fitness in order to enhance that of the other, i.e. to act in a *pro-social* manner; economists refer to preferences with *α* > 0 as *altruistic preferences* [26]. By contrast, an individual with *α* < 0 is willing to reduce own fitness in order to reduce that of the other, i.e. to act in an *anti-social* manner; economists refer to preferences with *α* < 0 as *spiteful* ones. Finally, fitness-maximizing individuals correspond to the special case *α* = 0. Although this class of preferences thus encompasses altruism, self-interest, and spite, I will simply refer to *α* as the *degree of altruism*.

A key insight delivered by the analyses of fitness game $\Gamma_{1}$ by Bester & Güth [21], Bolle [24] and Possajennikov [25] is that the evolutionary stability requires *α* to be of the same sign as *k*, the parameter that determines whether the strategies are strategic substitutes, strategic complements or strategically neutral. In order to show how one identifies ES degrees of altruism in interactions under complete information, I turn to the model of Alger & Weibull [17], which generalized the analysis of the same class of preference functions to any fitness game with a differentiable fitness function *w* such that there exists a unique and differentiable Nash equilibrium in any dyadic interaction; the analysis is further restricted to fitness functions *w* whereby an individual’s strategy has some effect on the other’s fitness, i.e. such that ∂*w*(*x*, *y*)/∂*y* ≠ 0 for all (*x*, *y*) ∈ *X*^2^ (the reason for this will be explained below).

Starting with the behavioural equilibrium in a dyad, let (x*(*α*, *β*), x*(*β*, *α*)) denote the Nash equilibrium strategy profile in a pair where one individual has degree of altruism *α* and the other has degree of altruism *β*. Recall that a Nash equilibrium strategy profile is such that neither individual would prefer to deviate to another strategy, given the other’s strategy, i.e.:

3.14 $$\begin{array}{rl} & x^{*}(\alpha ,\beta )\in \arg\max_{x\in X}\;w(x,y)+\alpha \cdot w(y,x)\\ \mathrm{and}\; & x^{*}(\beta ,\alpha )\in \arg\max_{y\in X}\;w(y,x)+\beta \cdot w(x,y). \end{array}\}$$

Since *w* is differentiable, the pair of first-order conditions is

3.15 $$\begin{array}{rl} & w_{1}(x^{*}(\alpha ,\beta ),x^{*}(\beta ,\alpha ))+\alpha \cdot w_{2}(x^{*}(\beta ,\alpha ),x^{*}(\alpha ,\beta ))=0\\ \mathrm{and}\; & w_{1}(x^{*}(\beta ,\alpha ),x^{*}(\alpha ,\beta ))+\beta \cdot w_{2}(x^{*}(\alpha ,\beta ),x^{*}(\beta ,\alpha ))=0, \end{array}\}$$

where the index 1 (respectively 2) indicates the partial derivative with respect to the first (respectively second) argument. Note that for altruism to have an effect on equilibrium strategies, *w*_2_( · ) ≠ 0 is necessary, and this explains why the analysis is restricted to such fitness functions, as announced above.

Turning now to the evolutionary stability analysis, let *α* denote the degree of altruism in the resident preference function and *β* that in the mutant preference function. Then, result 3.4 implies that for the function with degree of altruism *α* to be an ESPFC, it is necessary that

3.16 $$\begin{array}{r} w(x^{*}(\alpha ,\alpha ),x^{*}(\alpha ,\alpha ))\geq w(x^{*}(\beta ,\alpha ),x^{*}(\alpha ,\beta ))\;\forall \;\beta \in R, \end{array}$$

or, equivalently,

3.17 $$\alpha \in \arg\max_{\beta \in R}w(x^{*}(\beta ,\alpha ),x^{*}(\alpha ,\beta )).$$

Since *w* is differentiable, the necessary first-order condition is

3.18 $$\begin{array}{rl} & \left[w_{1}(x^{*}(\beta ,\alpha ),x^{*}(\alpha ,\beta ))\cdot x_{1}^{*}(\beta ,\alpha \right)\\ & {+w_{2}(x^{*}(\beta ,\alpha ),x^{*}(\alpha ,\beta ))\cdot x_{2}^{*}(\alpha ,\beta )]}_{|\beta =\alpha }=0, \end{array}$$

where again an index 1 (respectively 2) indicates the partial derivative with respect to the first (respectively second) argument. Recalling from (3.15) that *w*_1_(x*(*β*, *α*), x*(*α*, *β*)) = −*β* · *w*_2_(x*(*α*, *β*), x*(*β*, *α*)), equation (3.18) can be rewritten as

3.19 $$\begin{array}{rl} {}[ & -\beta \cdot w_{2}(x^{*}(\alpha ,\beta ),x^{*}(\beta ,\alpha ))\cdot x_{1}^{*}(\beta ,\alpha )\\ & {+w_{2}(x^{*}(\beta ,\alpha ),x^{*}(\alpha ,\beta ))\cdot x_{2}^{*}(\alpha ,\beta )]}_{|\beta =\alpha }=0.\; \end{array}$$

This reduces to the following simple equation, which in this setting is necessary for the function with degree of altruism *α* to be an ESPFC [17]:

3.20 $$\alpha \cdot x_{1}^{*}(\alpha ,\alpha )=x_{2}^{*}(\alpha ,\alpha ).$$

In fitness game $\Gamma_{1}$, simple calculations (i.e. solving (3.15) for *w* defined in (2.2)) lead to

3.21 $$x^{*}(\alpha ,\beta )=\frac{[2+(1+\alpha )k]m}{4-(1+\alpha )(1+\beta )k^{2}}.$$

In electronic supplementary material, §2, I reproduce the proof (found for different parameter values in [21] and [24–25]) that the unique ES degree of altruism is

3.22 $$\alpha^{*}=\frac{k}{2-k},$$

for any k∈(−1,0)∪(0,1), implying that α* has the same sign as *k*.

Equation (3.20) shows that the observability of the opponent’s preferences drives a wedge between fitness-maximizing preferences and ES preferences. Indeed, the right-hand side represents the effect that an individual’s preferences have on the *opponent’s* equilibrium strategy, and fitness-maximizing preferences (α* = 0) are ES if and only if this effect is nil ($x_{2}^{*}(0,0)=0$). More generally, the characterization in (3.20) unveils a connection between the qualitative nature of the fitness function *w* and the sign of the ES value of *α*.

**Result 3.5.**

(Alger & Weibull [17]) Let *w* be such that ∂*w*(*x*, *y*)/∂*y* ≠ 0 for all (*x*, *y*) ∈ *X*^2^. A preference function of the form (3.13) with *α* = α* is an ESPFC (within the set of all such functions) only if:

1. α* < 0 if the strategies are strategic substitutes;
2. α* > 0 if the strategies are strategic complements;
3. α* = 0 if the strategies are strategically neutral. ■

This result generates a clear prediction for the relationship between preferences on the one hand, and the specifics of the fitness function *w* on the other hand, where *w* presumably depends on the environment in which the population evolves. In environments with competition over resources, or where it pays off to free-ride on the other individual, evolutionary stability requires spiteful preferences. The reason is that spiteful preferences induce a commitment by the individual to engage in a higher extraction effort (or to free-ride), and this in turn induces a lower extraction effort (or a higher contribution) by the other; hence they can invade a population consisting of fitness-maximizers or altruistic individuals, as in the example that introduced this section. By contrast, in environments where teamwork is valuable, evolutionary stability requires altruistic preferences. The reason is that altruistic preferences induce a commitment by the individual to engage in higher effort in the team, and this in turn induces a higher effort by the other; for a small enough degree of altruism this enables them to invade a population consisting of fitness-maximizers or spiteful individuals.

The prediction reported in result 3.5 is testable if the researcher can measure whether individuals in the population at hand are willing to reduce own fitness in order to enhance that of the other (in which case *α* > 0), or rather to reduce it (in which case *α* < 0). If such direct measurement is impossible, the following comparison between the strategy that is employed by individuals in the population at hand and the ESS can be used as an indirect test:

**Result 3.6.**

Suppose that the fitness game Γ=⟨X,w⟩ is such that there is a unique ESS, denoted by *x*^ESS^, and that *w*(*x*, *x*) is increasing in *x* when evaluated around *x* = *x*^ESS^. Then in a population where a preference function of the form (3.13) with *α* = α* is an ESPFC (within the set of all such functions) and in which the (unique) Nash equilibrium strategy x*(α*, α*) is employed:

1. x*(α*, α*) < *x*^ESS^ if the strategies are strategic substitutes;
2. x*(α*, α*) > *x*^ESS^ if the strategies are strategic complements;
3. x*(α*, α*) = *x*^ESS^ if the strategies are strategically neutral. ■

If the fitness game Γ=⟨X,w⟩ is such that *w*(*x*, *x*) is decreasing in *x* when evaluated at *x* = *x*^ESS^, the reverse inequalities hold. Indeed, in fitness game $\Gamma_{1}$, one obtains

3.23 $$x^{*}(\alpha^{*},\alpha^{*})=\frac{m}{2-(1+\alpha^{*})k}.$$

Recalling that *x*^ESS^ = *m*/(2 − *k*) from (2.6), one indeed obtains x*(α*, α*) > *x*^ESS^ if *k* ∈ (0, 1), and x*(α*, α*) < *x*^ESS^ if *k* ∈ ( − 1, 0).

#### (i) Related models in the biology literature

Following McNamara *et al.* [4], a series of contributions in biology have examined the evolutionary stability of *negotiation rules*. This literature takes interest in fitness games whereby individuals engage in a series of interaction rounds that eventually lead to a ‘negotiated outcome’. Compared with the standard strategy evolution setting, where each individual is programmed to employ a certain strategy, here each individual is programmed with a response rule that specifies the strategy to play in response to the strategy used by the opponent in the previous round. This alternating process converges to a pair of strategies—the negotiated outcome—which the interactants then employ in the remaining rounds.

Like in the preference evolution literature, there is *a priori* no clear set of possible negotiation rules. McNamara *et al.* [4] and Taylor & Day [5] posit the following rule for an individual playing *x* in response to the opponent’s play of *y* in the previous round:

3.24 x=ρ−λ⋅y.

The evolving trait is the vector (*λ*, *ρ*), which represents the slope and the intercept. A population consisting of individuals with the rule (*λ*, *ρ*) = (0, *x*^ESS^) would play the ESS *x*^ESS^, and this rule is ES. However, there are also rules (*λ*, *ρ*) with *λ* ≠ 0 that are ES [4,5]. Note that the non-degenerate slope *λ* ≠ 0 implies that an individual’s behaviour is swayed by the opponent’s behaviour. The similarity with the non-nil effect of an individual’s preferences on the opponent’s behaviour in the model on the evolution of altruistic preferences under complete information (i.e. $x_{2}^{*}(\cdot ,\cdot )\neq 0$ in (3.20)) is thus clear. The following remark examines in greater detail the similarities and differences between the seminal models.

**Remark 3.7.**

An interesting parallel can be drawn between the response rule in (3.24) and the model analysed by Bester & Güth [21]. Recalling the fitness function that they posit (see (2.2)), an individual with altruistic preferences chooses a strategy *x* that maximizes the following expression, where *y* is the opponent’s strategy:

3.25 (m−x+ky)x+α⋅(m−y+kx)y.

The necessary (and sufficient) first-order condition for this maximization is

3.26 m−2x+ky+α⋅ky=0,

or

3.27 $$x=\frac{m}{2}+\frac{k(1+\alpha )}{2}y.\;$$

In other words, the best response of an individual with degree of altruism *α* to the opponent’s strategy is equivalent to the response rule examined by McNamara *et al.* [4] and Taylor & Day [5] (see (3.24)) for *ρ* = *m*/2 and *λ* = −(*k*(1 + *α*))/2. Hence, the system of necessary conditions for a Nash equilibrium strategy profile in a dyad with degrees of altruism (*α*, *α*′) at which Bester & Güth [21] evaluate fitness

3.28 $$\begin{array}{rl} & x^{*}(\alpha ,\alpha^{\prime })=\frac{m}{2}+\frac{k(1+\alpha )}{2}\cdot x^{*}(\alpha^{\prime },\alpha )\\ \mathrm{and}\; & x^{*}(\alpha^{\prime },\alpha )=\frac{m}{2}+\frac{k(1+\alpha^{\prime })}{2}\cdot x^{*}(\alpha ,\alpha^{\prime }) \end{array}\}$$

coincides with the system of equations that define the negotiated outcome in a dyad with response rules (*ρ*, *λ*), (*ρ*′, *λ*′) at which McNamara *et al.* [4] and Taylor & Day [5] evaluate fitness

3.29 $$\begin{array}{rl} & x^{*}((\rho ,\lambda ),(\rho^{\prime },\lambda^{\prime }))=\rho -\lambda \cdot x^{*}((\rho^{\prime },\lambda^{\prime }),(\rho ,\lambda ))\\ \mathrm{and}\; & x^{*}((\rho^{\prime },\lambda^{\prime }),(\rho ,\lambda ))=\rho^{\prime }-\lambda^{\prime }\cdot x^{*}((\rho ,\lambda ),(\rho^{\prime },\lambda^{\prime })) \end{array}\}$$

if *ρ* = *ρ*′ = *m*/2, *λ* = −*k*(1 + *α*)/2, and *λ*′ = −*k*(1 + *α*′)/2. This comparison highlights two differences between Bester & Güth [21] on the one hand, and McNamara *et al.* [4] and Taylor & Day [5] on the other hand. First, in the latter two papers both the slope and the intercept of the response rule are evolving traits, while in the former only the slope evolves. Second, they do not use the same fitness function. ■

This remark brings us to the contribution by Akçay *et al.* [6], which builds a nice bridge between the biology literature on the evolution of negotiation rules and the economics literature on preference evolution under complete information. In a model with the fitness function

3.30 $$w(x,y)=y^{1/2}-x^{2},$$

they consider preference functions of the form

3.31 $$u_{\beta }(x,y)=w(x,y)\cdot {\left(w(y,x)\right)}^{\beta },$$

and let *β* ≥ 0 be the evolving trait. They derive the best response of an individual with such preferences, they determine the conditions under which a negotiation phase would converge to Nash equilibrium in a complete information game between two individuals with such preferences, and they characterize the ES value of *β*. They further derive a result in a general model with generic but differentiable fitness and preference functions, such that in each dyad there exists a unique Nash equilibrium. This result can be described as follows:

**Result 3.8.**

(Akçay *et al.* [6]) Suppose that the fitness game Γ=⟨X,w⟩ is such that *w*(*x*, *x*) is increasing in *x*, and suppose that there is a unique ESS, denoted *x*^ESS^. Then in a population where a preference function of the form (3.31) with *β* = β* is an ESPFC and in which the (unique) Nash equilibrium strategy x*(β*, β*) is employed:

1. x*(β*, β*) > *x*^ESS^ if the strategies are strategic complements;
2. x*(β*, β*) = *x*^ESS^ if the strategies are strategically neutral. ■

The qualitative nature of this result is in line with that of a subset of the results found by Alger & Weibull [17] in the case of altruistic/spiteful preference functions (see result 3.6), an observation that would be expected in the light of the following remark.

**Remark 3.9.**

It is well known in economics that any preference ranking over items in an individual’s choice set that can be described by some preference function *u* can equally well be described by any positive monotone transformation of *u*. Taking the logarithm of the function posited by Akçay *et al.* [6] (see (3.31)), and defining

3.32 $$\tilde{u}(x,y)=\ln w(x,y)+\beta \cdot \ln w(y,x),$$

it is clear that this class of preference functions is qualitatively similar to the one adopted in the economics literature that built on Bester & Güth [21] (see (3.13)). ■

It is still an open question whether the qualitative nature of results 3.6 and 3.8 generalizes to other preference function classes. As mentioned earlier, it is *a priori* not clear which preference classes should be examined by modellers, a question that will be brought up again in the Discussion section.

### (c) Interactions under incomplete information

In this subsection, we will see that fitness-maximizers do prevail when interactions take place under incomplete information. By contrast to interactions that take place under complete information, in interactions where the individuals cannot observe each other’s preference function their behaviour cannot be swayed by the opponent’s preference function. However, an individual may still adapt behaviour to the *distribution* of preference functions present in the population. This is the assumption adopted in the analyses of preference evolution under incomplete information [19,20,27]. This section summarizes results by closely following the modelling assumptions of Alger & Weibull [27], for a reason that will become clear below. The set Θ of potential preferences is assumed to be less restrictive than above: it is the set of all continuous functions $u\;:\;X^{2}\to R$.

Let a population state s=(u,v,ε) be defined by the resident preference function u∈Θ, the mutant preference function v∈Θ, and the share ε of mutants. Under the same matching protocol as in the standard framework—i.e. that any individual faces a probability ε of being matched with a mutant—the criterion used in the literature is fitness evaluated at type-homogenous Bayesian Nash equilibrium (BNE) strategy profiles (below these will simply be referred to as equilibrium strategy profiles, or equilibria), defined as follows.

**Definition 3.10.**

In any state $s=(u,v,\varepsilon )\in \Theta^{2}\times (0,1)$, a strategy pair (x*, y*) ∈ *X*^2^ is a **type-homogenous Bayesian Nash equilibrium (BNE)** if

3.33 $$\begin{array}{rl} & x^{*}\in \arg\max_{x\in X}\;(1-\varepsilon )\cdot u(x,x^{*})+\varepsilon \cdot u(x,y^{*})\\ \mathrm{and}\; & y^{*}\in \arg\max_{y\in X}\;(1-\varepsilon )\cdot v(y,x^{*})+\varepsilon \cdot v(y,y^{*}). \end{array}\}$$

■

The first (respectively second) equation says that a resident (respectively a mutant) chooses a strategy that maximizes the expected value of the preference function *u* (respectively *v*), where the expectation is taken over the value that the preference function takes in a match with another resident (who plays x*), which realizes with probability 1−ε, and the value that it takes in a match with a mutant (who plays y*), which realizes with probability ε. Type-homogeneity means that all individuals with the same preference function (or preference type) use the same strategy. For example, consider again fitness game $\Gamma_{1}$, and assume that the resident preference function is of the form (3.13) with degree of altruism *α* while the mutant preference function is also of the form (3.13) but with degree of altruism *β* ≠ *α*. It is straightforward to verify that there then exists a unique type-homogenous BNE strategy profile, which depends on the share of mutants ε as follows:

3.34 $$\begin{array}{rl} & x^{*}(\varepsilon )=\frac{m[2+\varepsilon (\alpha -\beta )k]}{2[2-(1+\alpha )k+\varepsilon (\alpha -\beta )k]}\\ \mathrm{and}\; & y^{*}(\varepsilon )=\frac{m[2-(1-\varepsilon )(\alpha -\beta )k]}{2[2-(1+\alpha )k+\varepsilon (\alpha -\beta )k]}. \end{array}\}$$

Given some equilibrium strategy profile $\left(x^{*}(\varepsilon ),y^{*}(\varepsilon )\right)$ associated with population state s=(u,v,ε), define the equilibrium fitnesses of residents and mutants:

3.35 $$W_{u}(x^{*}(\varepsilon ),y^{*}(\varepsilon ),\varepsilon )=(1-\varepsilon )\cdot w(x^{*}(\varepsilon ),x^{*}(\varepsilon ))+\varepsilon \cdot w(x^{*}(\varepsilon ),y^{*}(\varepsilon ))$$

and

3.36 $$W_{v}(x^{*}(\varepsilon ),y^{*}(\varepsilon ),\varepsilon )=(1-\varepsilon )\cdot w(y^{*}(\varepsilon ),x^{*}(\varepsilon ))+\varepsilon \cdot w(y^{*}(\varepsilon ),y^{*}(\varepsilon )).$$

By contrast to the analyses of interactions under complete information, the typical approach for interactions under incomplete information consists in minimally constraining the set of possible preference functions, Θ. In particular, it turns out that it is possible to derive general results even for settings in which there are states s=(u,v,ε) with multiple equilibria.

**Definition 3.11.**

(Alger & Weibull [27]) A preference function u∈Θ is **evolutionarily stable under incomplete information (ESI)** against a function v∈Θ if there exists an $\bar{\varepsilon }>0$ such that $W_{u}(x^{*}(\varepsilon ),y^{*}(\varepsilon ),\varepsilon )>W_{v}(x^{*}(\varepsilon ),y^{*}(\varepsilon ),\varepsilon )$ in all Nash equilibria $\left(x^{*}(\varepsilon ),y^{*}(\varepsilon )\right)$ in all states s=(u,v,ε) with $\varepsilon \in (0,\bar{\varepsilon })$. A preference function *u* is an **evolutionarily stable preference function under incomplete information (ESPFI)** if it is ESI against all preference functions *v* ≠ *u* in Θ. ■

To illustrate the analytical challenge that this setting presents, focus momentarily on a setting where in each state $s=(u,v,\varepsilon )\in \Theta^{2}\times (0,1)$ there exists a unique equilibrium strategy profile. In such a setting, the score function is:

3.37 $$\begin{array}{rl} S_{u,v}(\varepsilon ) & =(1-\varepsilon )\cdot [w(x^{*}(\varepsilon ),x^{*}(\varepsilon ))-w(y^{*}(\varepsilon ),x^{*}(\varepsilon ))]\\ & \;+\varepsilon \cdot [w(x^{*}(\varepsilon ),y^{*}(\varepsilon ))-w(y^{*}(\varepsilon ),y^{*}(\varepsilon ))]. \end{array}$$

Clearly, the score function is not necessarily linear in ε (not even in the simple fitness game $\Gamma_{1}$, as can be readily inferred from the expressions in (3.34)). What is worse, without further assumptions the score function may even be discontinuous, since the equilibrium strategy profile may vary discontinuously with ε. To see this, recall the Stag hunt fitness game $\Gamma_{2}$ with fitness function *w*(*x*, *y*) = *xyR* + 1 − *x* (see (2.3)), and assume that the set of strategies is {0, 1/4, 1}, i.e. an individual can either choose the pure strategy *H* with certainty, choose the pure strategy *S* with certainty, or use some randomization device that leads to *S* with probability 1/4 and to *H* with probability 3/4 (in the hunting story, perhaps the hunter has decided to go for the stag if and only if (s)he sees a snake on the path, an event that happens with probability 1/4). Consider preference functions of the form

3.38 u(x,y)=xy(R+K)+1−x,

where K∈R can be interpreted, for example, as the joy (if *K* > 0) or the sadness (if *K* < 0) experienced when killing a stag. Suppose that *R* = 2 and that the resident preference function has *K* = 2. In a population consisting entirely of residents, there are three Nash equilibrium strategy profiles: the two pure strategy profiles (0, 0) and (1, 1), as well as the mixed-strategy equilibrium profile (1/4, 1/4).^7^ Suppose now that a share ε of the population strongly dislike killing stags, and has the mutant preference function of the form (3.38) with *K* = −4. Then a BNE satisfies

3.39 $$\begin{array}{rl} & x^{*}\in \arg\underset{x\in \{0,1/4,1\}}{\max}\;(1-\varepsilon )\cdot (4xx^{*}+1-x)\\ & \;\;\;\;\;+\varepsilon \cdot (4xy^{*}+1-x)\\ \mathrm{and}\; & y^{*}\in \arg\underset{y\in \{0,1/4,1\}}{\max}\;(1-\varepsilon )\cdot (-2yx^{*}+1-y)\\ & \;\;\;\;\;+\varepsilon \cdot (-2yy^{*}+1-y). \end{array}\}$$

Clearly, for the mutants the pure strategy *H* is strictly dominant, meaning that they strictly prefer it to the other two strategies, independent of the opponent’s strategy. Hence, in any BNE in this population, the mutants use strategy y* = 0, and the residents choose some strategy such that

3.40 $$\begin{array}{r} x^{*}\in \arg\max_{x\in \{0,1/4,1\}}\;(1-\varepsilon )\cdot (4xx^{*}+1-x)+\varepsilon \cdot (1-x). \end{array}$$

This fixed-point problem admits the solution x* = 0 for any ε∈(0,1) and the solution x* = 1 for any ε∈(0,3/4], implying that (x*, y*) = (1, 0) and (x*, y*) = (0, 0) are both BNE for small values of ε. However, for any ε>0, a resident is no longer indifferent between *H* and *S* when all the other residents play *x* = 1/4, since (1−ε)4/4<1. Hence (x*, y*) = (1/4, 0) is not a BNE, and there is thus a discontinuity at ε=0: while residents may use the mixed strategy 1/4 when there are no mutants around, they no longer do so as soon as the mutant preference function at hand is present in the population.

The potential existence of multiple equilibria together with potential discontinuities in the set of BNE introduces a sharp contrast with the linearity in ε of the score functions under strategy evolution (2.4) and under preference evolution under complete information (3.12), which implied that analysis of the score function at ε=0 was sufficient to check evolutionary stability (recall results 2.6 and 3.4). In spite of these challenges, there are conditions that render general analysis possible, even for settings with multiple equilibria. In particular, several authors have shown that the fitness-maximizing preference function is ES.

Prior to stating one such result, one additional issue needs to be addressed, however. Recall that the goal here is to minimally constrain the set of possible preference functions, Θ. In particular, Θ contains several functions that give rise to the same strategy choices (recall remark 3.9), and hence that in general there will be no preference function that is ESI against all the other preference functions (since residents must obtain a *strictly* higher average fitness than mutants for it to be ES, see definition 3.11). The following definition of *behavioural alikes* addresses this issue.

**Definition 3.12.**

Let *X*_0_ be the set of type-homogenous Nash equilibria in a population consisting solely of fitness-maximizers. A preference function *f* is a **behavioural alike** to fitness-maximizers if there exists some *x*_0_ ∈ *X*_0_ and some *z* ∈ *X* such that $z\in \arg\max_{x\in X}w(x,x_{0})$ and $z\in \arg\max_{x\in X}f(x,x_{0})$. ■

In words (and somewhat loosely) a behavioural alike to fitness-maximizers is a preference type that would be willing to play a strategy *z* (perhaps different from *x*_0_) that the fitness-maximizer would also be willing to play, given that the opponent plays some *x*_0_ ∈ *X*_0_. The following result identifies sufficient conditions for the fitness-maximizing preference function to be ES. This result is found in [28] (it is a slight variation of the result as stated in the earlier article [27], the difference stemming from a slight difference in how behavioural alikes are defined; the core of the result is not affected, however).

**Result 3.13.**

If the strategy set *X* is compact and convex, and all the preference functions in Θ as well as the fitness function *w* are continuous, then the fitness-maximizing preference function (see (3.1)) is ESI against any preference function that is not its behavioural alike. ■

The topological properties stated in the result ensure that the correspondence, which to each population state $s=(u,v,\varepsilon )\in \Theta^{2}\times (0,1)$ associates the set of equilibrium strategy profiles, is upper-hemicontinuous in ε. Hence, even if the introduction of an infinitesimal share of mutants sways the equilibrium strategy of the residents away from the equilibrium strategy played in the absence of mutants, the ‘new’ equilibrium strategy is arbitrarily close to some strategy that the fitness-maximizers could have played in the absence of mutants. Continuity of the fitness function then implies that any mutant that is not a behavioural alike to fitness-maximizers obtains a strictly lower equilibrium fitness than the fitness-maximizers.

Ok & Vega-Redondo [19] adopt similar topological properties, and they show that fitness-maximizers are robust to the entry of non-fitness-maximizers even in finite but large enough populations. By contrast, in small populations the entry of mutants makes the resident fitness-maximizers shift their strategy away from any strategy they would have played in the absence of mutants in many fitness games, and the result no longer holds (this is reminiscent of the fact that a strategy that is ES in infinite populations is not necessarily ES in finite populations [29]).

Why should evolutionary biologists take interest in result 3.13? After all, the prediction is not surprising: under incomplete information fitness-maximizers prevail in a panmictic setting. I would argue that the result is valuable because it solves a dilemma inherent to fitness games with multiple ESSs. Indeed, for such fitness games, analysis under the assumption that selection operates at the level of strategies delivers no clear prediction. By contrast, analysis under the assumption that selection operates at the level of preferences predicts that one particular preference function stands out as being viable from an evolutionary perspective.^8^

## 4. Strategy and preference evolution in the presence of relatedness

All of the results summarized above were derived in the panmictic setting [10], where the probability of being matched with a mutant is the same for residents and mutants, which implies that as the share of mutants tends to 0 the probability that a mutant is matched with another mutant tends to 0 as well. Relatedness means that a rare mutant is more likely to be matched with another mutant than a resident is to be matched with a mutant [7,8,30]. Relatedness arises in naturally structured populations [31] and is part of the environment of evolutionary adaptation of the human lineage [32]. It has been shown to depend on a number of factors, such as migration rates, and even the strategies present in the population (e.g. [13]). However, here I adopt the reduced-form approach originally proposed by Bergstrom [33] (the term commonly used in the economics literature is assortativity of the matching process rather than relatedness).

**Definition 4.1.**

For any given resident preference function *u* and mutant preference function *v*, and share ε∈(0,1) of mutants, let Pr(v|u,ε) denote the probability that a resident is matched with a mutant, and Pr(v|v,ε) the probability that a mutant is matched with another mutant. Assuming that the conditional probability functions are continuous in ε, and that the probability that a mutant is matched with another mutant tends to some number *r* ∈ [0, 1] as the share of mutants tends to 0, i.e.

4.1 $$\lim_{\varepsilon \to 0}\text{Pr}(v|v,\varepsilon )=r,$$

the number *r* measures *relatedness* between interactants. ■

The analyses summarized above correspond to the special case Pr[v|u,ε]=Pr[v|v,ε]=ε for all (u,v,ε), and thus *r* = 0.

Prior to examining preference evolution, it is worth noting that the definition of an ESS is readily extended to encompass relatedness (an early analysis can be found in [34]):

**Definition 4.2.**

Consider a population in which individuals are randomly matched into pairs to interact according to the fitness game Γ=⟨X,w⟩. A strategy *x* ∈ *X* is **evolutionarily stable (ES)** against strategy *y* ∈ *X*, *y* ≠ *x*, if there exists ${\bar{\varepsilon }}_{y}\in (0,1)$ such that for all $\varepsilon \in (0,{\bar{\varepsilon }}_{y})$:

4.2 Pr(x|x,ε)⋅w(x,x)+Pr(y|x,ε)⋅w(x,y)>Pr(x|y,ε)⋅w(y,x)+Pr(y|y,ε)⋅w(y,y).

And *x* is an **ESS** if it is ES against all *y* ∈ *X*, *y* ≠ *x*. ■

Hence, in a setting with relatedness *r* ∈ [0, 1], result 2.6 generalizes to [27]:

**Result 4.3.**

If Pr(*x*|*x*, *ε*) − Pr(*x*|*y*, *ε*) = *r* for all ε ∈ (0,1), then:

1. If *w*(*x*, *x*) > *w*(*y*, *x*) + *r* · [*w*(*y*, *y*) − *w*(*y*, *x*)], then *x* is ES against *y*.
2. If *w*(*x*, *x*) = *w*(*y*, *x*) + *r* · [*w*(*y*, *y*) − *w*(*y*, *x*)], then *x* is ES against *y* if and only if *w*(*x*, *y*) > *w*(*y*, *y*) + *r* · [*w*(*y*, *y*) − *w*(*y*, *x*)].
3. If *w*(*x*, *x*) < *w*(*y*, *x*) + *r* · [*w*(*y*, *y*) − *w*(*y*, *x*)], then *x* is not ES against *y*. ■

**Remark 4.4.**

Recalling remark 2.2 for the panmictic case, the model in definition 4.2 can again be seen as the special case of the general model by Lehmann & Rousset [13], in which there is relatedness *r*, haploidy, and weak selection. In this case the invasion fitness (their eqn (2)) reduces to [(1 − *r*)*w*(*y*, *x*) + *rw*(*y*, *y*)]/*w*(*x*, *x*), and the condition for *x* to be uninvadable (their eqn (2.1)) is equivalent to (1 − *r*)*w*(*y*, *x*) + *rw*(*y*, *y*) ≤ *w*(*x*, *x*), a condition that is necessary for *x* to be ESS in the present setting. ■

Turning now to a summary of the results for preference evolution, the distinction between complete and incomplete information is still called for.

### (a) Interactions under complete information

Starting with interactions under complete information and examining, as above, preference functions of the form (3.13) whereby an individual attaches some weight α∈R to the other’s individual fitness, the definition of an ES preference function under complete information (see definition 3.3) readily generalizes to encompass relatedness by replacing 1−ε and ε by the appropriate conditional matching probabilities in (3.11), to obtain:

4.3 $$\text{Pr}(u|u,\varepsilon )\cdot w(x^{*},x^{*})+\text{Pr}(v|u,\varepsilon )\cdot w(\hat{x},\hat{y})>\text{Pr}(u|v,\varepsilon )\cdot w(\hat{y},\hat{x})+\text{Pr}(v|v,\varepsilon )\cdot w(\tilde{x},\tilde{x}).$$

The score function in (3.12) thus generalizes to:

4.4 $$\begin{array}{rl} S_{u,v}(\varepsilon ) & =\text{Pr}(u|u,\varepsilon )\cdot w(x^{*},x^{*})+\text{Pr}(v|u,\varepsilon )\cdot w(\hat{x},\hat{y})\\ & \;-\text{Pr}(u|v,\varepsilon )\cdot w(\hat{y},\hat{x})-\;\text{Pr}(v|v,\varepsilon )\cdot w(\tilde{x},\tilde{x}). \end{array}$$

Recall that differentiability of this function facilitates analysis, since it is then sufficient to examine the value (and sometimes the derivative) of *S*_*u*,*v*_ at ε=0 to establish whether *u* is ESC against *v*. Such differentiability obtains if the conditional probability functions are differentiable. Positing such differentiability, result 3.5 generalizes to:

**Result 4.5.**

(Alger & Weibull [17]) In a population where the matching process entails relatedness *r* ∈ [0, 1], and the conditional probability functions are differentiable in ε, a preference function of the form (3.13) with *α* = α* is an ESPFC only if:

1. α* < *r* if the strategies are strategic substitutes;
2. α* > *r* if the strategies are strategic complements;
3. α* = *r* if the strategies are strategically neutral. ■

The complete information setting may be particularly well suited to represent interactions between relatives, since relatives often have the opportunity to observe each other’s behaviours for many years. Given that the value of *α* determines how willing individuals are to act generously towards the other, the result suggests that evolution may have led to variation in the degree of intra-family generosity across different regions of the world. Indeed, in our evolutionary past the qualitative nature of the fitness game in any given region may have depended on the local ecological conditions. For example, in Arctic regions whale hunting was common, and such hunting arguably involves strategic complementarities. By contrast, in agricultural societies, food production would arguably have been a game in which production efforts were strategic substitutes. Furthermore, even for a given category of fitness game (i.e. whether strategies are strategic substitutes, complements or neutral), the specifics of the fitness game are expected to matter. For example, in the production-and-sharing fitness game studied in [35], where the strategies are strategic substitutes, the ES degree of altruism is found to be lower in harsh than in generous environments, for a given relatedness *r*.

### (b) Interactions under incomplete information

Turning now to interactions under incomplete information, a straightforward generalization of the panmictic setting examined above is possible. Inserting the conditional probabilities into the system of best-response equations (3.33) in definition 3.10 of a BNE,

4.5 $$\begin{array}{rl} & x^{*}\in \arg\max_{x\in X}\;\text{Pr}(u|u,\varepsilon )\cdot u(x,x^{*})+\text{Pr}(v|u,\varepsilon )\cdot u(x,y^{*})\\ \mathrm{and}\; & y^{*}\in \arg\max_{y\in X}\;\text{Pr}(u|v,\varepsilon )\cdot v(y,x^{*})+\text{Pr}(v|v,\varepsilon )\cdot v(y,y^{*}), \end{array}\}$$

and into the equilibrium fitnesses of residents and mutants (see (3.35) and (3.36)),

4.6 $$\begin{array}{r} W_{u}(x^{*},y^{*},\varepsilon )=\text{Pr}(u|u,\varepsilon )\cdot w(x^{*},x^{*})+\text{Pr}(v|u,\varepsilon )\cdot w(x^{*},y^{*}) \end{array}$$

and

4.7 $$\begin{array}{r} W_{v}(x^{*},y^{*},\varepsilon )=\text{Pr}(u|v,\varepsilon )\cdot w(y^{*},x^{*})+\text{Pr}(v|v,\varepsilon )\cdot w(y^{*},y^{*}), \end{array}$$

the definition of an ES preference function under incomplete information applies as is (see definition 3.11).

As was the case for preference evolution under incomplete information in the panmictic setting, the goal is to impose minimal restrictions on the set of potential preferences. The simple fitness-maximizing preference function (see (3.1)) is no longer ES, however. Instead, the analyses in [27,28] reveal that evolution favours *Homo moralis* preferences:

**Definition 4.6.**

An individual is a ***Homo moralis*** if his/her preference function is of the form

4.8 $$u_{\kappa }(x,y)=(1-\kappa )\cdot w(x,y)+\kappa \cdot w(x,x),$$

for some *κ* ∈ [0, 1], his/her degree of morality. ■

While it was the mathematical analysis that led to the ‘discovery’ of this preference class, the name *Homo moralis* was inspired by the second term in (4.8), which can be interpreted as a concern for universalization, reminiscent of Kant’s reasoning [36]: what would happen (to the individual’s fitness) if the individual’s strategy was universalized? The first term being the individual’s fitness given own and opponent’s actual strategies, the *Homo moralis* preference function can be thought of as representing a form of partial Kantian moral concern (see also [37] for a similar ‘as if’ interpretation, in a model with strategy evolution for interactions between siblings).

As before, it is necessary to precisely define behavioural alikes (recall definition 3.12).

**Definition 4.7.**

Let *X*_*r*_ be the set of type-homogenous Nash equilibria in a population consisting solely of *Homo moralis* with degree of morality *κ* = *r*. A preference function *f* is a **behavioural alike** to such *Homo moralis* if there exists some *x*_*r*_ ∈ *X*_*r*_ and some *z* ∈ *X* such that $z\in \arg\max_{x\in X}(1-r)\cdot w(x,x_{r})+r\cdot w(x,x)$ and $z\in \arg\max_{x\in X}f(x,x_{r})$. ■

In words, a behavioural alike to a *Homo moralis* with a degree of morality *κ* = *r* is a preference type that would be willing to play a strategy that such a *Homo moralis* would also be willing to play, given that the opponent uses some type-homogenous BNE strategy in a monomorphic population of such *Homo moralis*, *x*_*r*_ ∈ *X*_*r*_. Then [28] shows the following result (a slight variation can be found in [27], where the main difference is a slight and unimportant difference in the definition of behavioural alikes, already referred to above).

**Result 4.8.**

If the strategy set *X* is compact and convex, and all the preference functions in Θ as well as the fitness function *w* are continuous, then the *Homo moralis* preference function with degree of morality equal to the coefficient of relatedness, *κ* = *r* (see (4.6)) is ESI against any preference function that is not its behavioural alike. ■

A population of *Homo moralis* resists entry by mutants because their preferences make them select a strategy that pre-empts entry by mutants. To see this, note first that the average fitness of vanishingly rare mutants (see (4.7)), who play some strategy, say *z*, tends to the following value as ε tends to 0:^9^

4.9 $$(1-r)\cdot w(z,x_{r})+r\cdot w(z,z),$$

where *x*_*r*_ is some Nash equilibrium strategy in a monomorphic population consisting of *Homo moralis*:

4.10 $$x_{r}\in \arg\max_{x\in X}(1-r)\cdot w(x,x_{r})+r\cdot w(x,x).$$

A mutant preference type that is not a behavioural alike to *Homo moralis* with degree of morality *κ* = *r* necessarily plays a strategy that does *not* belong to the set $\arg\max_{x\in X}(1-r)\cdot w(x,x_{r})+r\cdot w(x,x)$. Hence, (1 − *r*) · *w*(*z*, *x*_*r*_) + *r* · *w*(*z*, *z*) < (1 − *r*) · *w*(*x*_*r*_, *x*_*r*_) + *r* · *w*(*x*_*r*_, *x*_*r*_) = *w*(*x*_*r*_, *x*_*r*_). In other words, the average fitness of vanishingly rare mutants is strictly smaller than the average fitness of residents, which is arbitrarily close to *w*(*x*_*r*_, *x*_*r*_). In sum, a population of *Homo moralis* resists entry by mutants because their preferences make them select a strategy that maximizes the average fitness of vanishingly rare mutants, given that the residents play this strategy.

#### (i) Related models in the biology literature

Biologists will of course have recognized Hamilton’s rule [7,8] in the results presented in this section (expressed at the relevant level of selection [38], i.e. either at the level of strategies, or at the level of preferences). One may therefore wonder whether analysis of preference evolution brings fundamental new insights to the rich literature that followed in Hamilton’s footsteps. I argue that the answer is positive for two reasons.

First, economists propose a rigorous and general analysis of behavioural Nash equilibria that is absent from the biology literature. As shown above, this has made it possible to tackle new questions such as: ‘What can be said about ES preferences when there are multiple Nash equilibria?’, and ‘What can be said about ES preferences when the set of potential preference functions is the set of all continuous functions?’

Second, and more speculatively, there may be an interesting parallel to be drawn between, on the one hand, the preference evolution literature, and on the other hand the literature that asks whether individuals who use an ESS can be viewed *as if* they are maximizing some goal function (recently reviewed by Lehmann & Rousset [13]; see also [37] for an early such analysis in the economics literature). Specifically, the idea is that this parallel may perhaps shed additional light on the comparison of the gene-centred and the actor-centred views, discussed at length in [13] and in works surveyed therein.

The argument rests on a comparison between the altruistic preference function, defined in (3.13), and the *Homo moralis* preference function, defined in (4.8). An individual with an altruistic preference function evaluates the consequences of his/her strategy on own individual fitness as well as on the interactant’s individual fitness. This is reminiscent of the actor-centred view, which aggregates the effects of the individual on the others. An individual with a *Homo moralis* preference function evaluates the consequences of his/her strategy on own individual fitness as well as on what his/her own individual fitness would be if, hypothetically, the interactant were to use the same strategy instead of the one (s)he is actually using. This is reminiscent of the gene-centred view, which aggregates the effects of others’ behaviours on the individual’s fitness.

The point is that the literature on preference evolution under incomplete information shows that *Homo moralis* preferences are more robust than altruistic preferences, in the following sense. Recall from result 4.8 that *Homo moralis* preferences with degree of morality *κ* = *r* are ES. It turns out that there are fitness games for which the set of Nash equilibria in a monomorphic population of altruists with degree of altruism *α* = *r* coincides with the set of Nash equilibria in a monomorphic population of *Homo moralis* with degree of morality *κ* = *r*. For such games neither preference function is ES against the other, and they are both ES against preference functions that are not behavioural alikes of *Homo moralis*. However, there are also fitness games in which the said set of Nash equilibria do not coincide. In such games, only *Homo moralis* preferences with degree of morality *κ* = *r* are ES, while altruistic preferences with degree of altruism *α* = *r* may fail to be ES. I will now show that the former situation arises in fitness game $\Gamma_{1}$ while the latter case arises in fitness game $\Gamma_{2}$.

In fitness game $\Gamma_{1}$, when interacting with an individual who uses strategy *y*, a *Homo moralis* chooses a strategy that satisfies the necessary first-order condition for *x* to maximize *u*_*κ*_(*x*, *y*):

4.11 (1−κ)(m+ky−2x)+κ(m+2kx−2x)=0.

This implies that there is a unique Nash equilibrium strategy in a monomorphic population consisting of *Homo moralis* with degree of morality *κ* = *r*, which is the unique solution to the equation

4.12 (1−r)(m+kx−2x)+r(m+2kx−2x)=0.

In this fitness game, an individual with altruistic preferences chooses a strategy that satisfies the necessary first-order condition for *x* to maximize *u*_*α*_(*x*, *y*),

4.13 m+ky−2x+αky=0,

implying that the unique Nash equilibrium strategy in a monomorphic population consisting of such altruists with degree of altruism *α* = *r* solves

4.14 m+kx−2x+rkx=0.

Equations (4.12) and (4.14) both yield *x* = *m*/[2 − (1 + *r*)*k*]: the set of equilibria in both monomorphic populations being the same, both preference functions are ES against preference functions that are not behavioural alikes of *Homo moralis* with degree of morality *κ* = *r*.

Turning now to fitness game $\Gamma_{2}$, a strategy *x*_*α*_ is a symmetric Nash equilibrium in a monomorphic population consisting of altruists with degree of altruism *α* if and only if an individual would not like to deviate to any other strategy, given that the other plays *x*_*α*_:

4.15 $$\begin{array}{rl} & \left(1+\alpha )[x_{\alpha }^{2}R+1-x_{\alpha }]\geq (1+\alpha )xx_{\alpha }R+1-x+\alpha (1-x_{\alpha }\right)\\ & \;\forall \;x\in [0,1]. \end{array}$$

For any value of *α* ∈ [0, 1], there are three such equilibrium strategies: {0, 1/[(1 + *α*)*R*], 1}. Turning now to *Homo moralis* preferences, a strategy *x*_*κ*_ is a symmetric Nash equilibrium in a monomorphic population consisting of *Homo moralis* with degree of morality *κ* if and only if

4.16 $$\begin{array}{rl} & x_{\kappa }^{2}R+1-x_{\kappa }\geq (1-\kappa )(xx_{\kappa }R+1-x)+\kappa (x^{2}R+1-x)\\ & \;\forall \;x\in [0,1]. \end{array}$$

Here the set of such equilibrium strategies is {0, 1} if and only if *κ* ∈ [0, 1/*R*], and {1} otherwise. In this fitness game, the *Homo moralis* preference function with *κ* = *r* is ES (as per result 4.8). By contrast, the altruistic preference function with *α* = *r* is not. To see this, assume that this is the resident preference function and consider a mutant preference function that induces the mutants to choose *y* = 1. Then in one of the BNE (see (4.5)) residents play the mixed strategy

4.17 $$x=\frac{1-\mathrm{Pr}(v|u,\varepsilon )(1+\alpha )R}{\mathrm{Pr}(u|u,\varepsilon )(1+\alpha )R}.$$

Clearly, the mutants obtain a strictly higher average fitness than such resident altruists, when these use this mixed strategy. In this case, the altruistic preferences with *α* = *r* are thus not ES, while *Homo moralis* preferences are (as per result 4.8).

## 5. Discussion

The first contributions to the literature on the evolution of preferences by natural selection extended the concept of evolutionary stability from the level of strategies [1] to the level of preferences guiding the choice of strategy, an approach that is sometimes referred to as *indirect evolution* [21], since evolution then operates on strategies only indirectly, by ‘delegating’ the strategy choice to the individual. Several novel insights were delivered by these contributions, as summarized above. This literature is arguably still in its infancy, and I here discuss some possible future paths.

To begin, some readers may wonder: *is there really a deep difference between strategy evolution and preference evolution?* After all, and as highlighted in this article, it is typically possible to reformulate preference evolution as evolution of response rules. I would make the case that there is a fundamental difference, however. Strategies are mere descriptions of behaviour. Preferences are expressed within individuals as the result of some process that may involve reasoning, emotions, hormones and/or other neurobiological mechanisms, and that may respond to the stimuli and information the individual receives. Hence, individuals are guided by their preferences even in completely novel situations, implying that their behaviour may change when, for instance, a hitherto unknown preference function appears in the population. Furthermore, some preference classes present the advantage of lending themselves to psychological interpretation. For example, one possible interpretation of an individual with altruistic preferences of the form (3.13) with a positive degree of altruism *α* > 0 is that (s)he has emotions that are swayed by the fitness of the person with whom (s)he interacts: the better off is the opponent, the happier (s)he gets. By contrast, an individual *Homo moralis* preferences of the form (4.8) with a positive degree of morality *κ* > 0 would not react to information about the opponent’s fitness: (s)he instead evaluates different courses of action by taking into account what own fitness would be if—hypothetically—the course of action was universalized to all the interactants. Experimental research shows that these motivations can be distinguished empirically [39,40].

These observations further suggest three possible future research paths.

First, over the past few decades the behavioural economics literature has proposed and examined a wealth of preference classes to explain observed behaviours in social interactions: altruism [26], warm glow [41], a preference for conformity [42], a preference for reciprocity [43–46], inequity aversion [47,48], guilt aversion [49,50] and image concerns [51,52]. These preference classes were inspired mostly by research in psychology and sociology. Building the interdisciplinary bridge one step further by evaluating the evolutionary stability properties of these preferences classes would be interesting; note that this is related to the suggestion made by Sober & Wilson [53] that evolutionary viability of psychological motives behind unselfish behaviours ought to be examined. It should be noted in this context that *Homo moralis* preferences [27], studied in this article, are novel to behavioural economics: the theory of preference evolution has thus contributed to economics through the discovery of a hitherto unstudied preference class, and future analyses may lead to further similar discoveries.

Second, the theory of preference evolution may unveil ultimate drivers of the aforementioned preference classes (besides altruistic and *Homo moralis* preferences, already extensively studied). A question of particular interest is whether there may be stable polymorphisms—populations in which several preference classes co-exist—and if so, which factors are expected to affect the stable distribution of preferences. Such theories may help explain observed heterogeneity both within and between populations in survey and experimental data [54–56].

Third, researchers working with models of preference evolution must make assumptions on the set of potential preference functions. In reality, however, the set of potential preference functions available for a given organism may be determined by physiological constraints. An open question is thus whether findings on the neurobiology of our species would help reduce this set [57]. Such an approach has already been used in the theoretical literature on the evolution of preference functions that govern choices in decision situations other than social interactions (e.g. [58,59]).

Readers may also ask: *how realistic is the process by which individuals are matched together in preference evolution models that extend the standard evolutionary game theory model?* In particular, are the results found under this assumption robust to the extension to other matching processes? Two nascent paths can be mentioned in this context.

First, the model of preference under incomplete information found in [27,28] has been incorporated by Alger *et al.* [60] into a standard island model [61], in which the population is structured into groups between which there is limited migration. This approach allowed the researchers to distinguish between preference functions defined over fitness on the one hand and preference functions defined over material payoffs on the other hand. Arguably, the preference function defined over material payoffs that is found to be uninvadable in [60] is more relevant for social scientists who seek to estimate the preferences of individuals by way of observing their behavioural responses to trivial material payoff consequences, such as in the experimental economics literature [39,44,62–64]. This function combines material self-interest and a Kantian moral concern *à la* *Homo moralis* expressed at the material payoff level, with a third component, which can be interpreted as altruism/spite towards the opponent, again at the material payoff level. While this function thus differs from the *Homo moralis* function, the *Homo moralis* preference function is still uninvadable when defined over fitnesses rather than material payoffs, thus providing one first robustness test. It remains to be seen which preference functions—or distributions over preference functions—would resist the invasion of mutants in models with more sophisticated modelling of the migration decisions, such as in [65], for example.

Second, individuals are typically free to choose with whom they interact. Such active partner choice is known to matter for the evolution of cooperative strategies [66]. How would it affect the evolution of preference functions? One possible formalization is provided by Hopkins [67], in a model with altruistic preference functions where individuals differ in their ability to understand the mental processes of others.

Readers may further ask: *if preferences emanate from mental and neurobiological processes, is it reasonable to assume that one can observe others’ preferences?* In the model proposed by Heller & Mohlin [68], this issue—reminiscent of the well-known ‘mimicry’ issue in biology—is addressed by examining the co-evolution of preferences and the ability to deceive others about preferences and intentions. The extensive work on the commitment role that emotions may have played in our evolutionary past, and the concomitant ability to signal (e.g. through anger) and also detect such emotions (see, e.g. [69]), may perhaps also inspire formal work on emotions that can be incorporated into the theory of preference evolution.

The definition of an ESS [1] provided a key tool for theorists to model ultimate drivers of behaviour in social interactions. Adding the idea that nature delegates the strategy choice to the individuals by way of equipping them with preferences over strategies [2,3], arguably brings the theory closer to reality. Although the literature has already delivered many insights, most of the work on evolutionarily viable preferences undoubtedly still lies ahead of us. I hope that this article has underlined the fundamental role played by the bridges built between the models of biologists and economists, both in the past and in the future.

## Data Availability

The data are provided in electronic supplementary material [70].

## Endnotes

1 Personal fitness measures the individual’s reproductive success, i.e. the number of offspring. Note that if the transmission is cultural, then these are not the biological but the cultural offspring.

2 McNamara & Leimar [11] also view *w*(*y*, *x*) as a proxy of invasion fitness.

3 The framework even applies to both finitely and infinitely repeated games, in which a strategy specifies which action to undertake as a function of the *history of play* [12]; due to the complex notation required to rigorously define repeated games and to space restrictions, however, I will not explicitly study these games here.

4 Note that fitness games for which *∂*^2^ *w*(*x*, *y*)/(∂*x*∂*y*) does not have the same sign for all (*x*, *y*) ∈ *X*^2^ fall in neither category.

5 Note that the score function is different from the selection gradient, which is the derivative with respect to the trait value; the slope of the score function instead indicates whether the difference between the average fitness earned by residents and that earned by mutants increases or decreases as the share of mutants ε increases. The selection gradient (for fitness game $\Gamma_{1}$) will be found in equation (2.6).

6 Researchers who have adopted this definition do not necessarily believe that individuals in real life do play some Nash equilibrium; however, it is a useful first approach, and the definition can be readily adapted to other equilibrium notions.

7 To see this, note that an individual is indeed indifferent between the two pure strategies *S* and *H*, given that the opponent chooses *S* with probability 1/4, since (*R* + *K*)/4 = 4/4 = 1. Such indifference is necessary and sufficient for the individual to be willing to randomize between *H* and *S*.

8 Note, however, that for such fitness games the set of Nash equilibria in a population consisting of fitness-maximizers does not necessarily coincide with the set of ESSs. For example, in the Stag hunt game with the (convex) set of strategies *X* = [0, 1] and parameter *R* = 3 in the fitness function (2.3), the set of symmetric Nash equilibrium strategies is {0, 1/3, 1}, while the set of ESSs is {0, 1}. This can be understood by recalling result 2.6: suppose there is strategy evolution, that the resident strategy is *x* = 1/3 and the mutant strategy is *y* = 0; then *w*(*x*, *x*) = *w*(*y*, *x*) = 1, but *w*(*x*, *y*) = 2/3 < 1 = *w*(*y*, *y*), and hence *x* is not ES against *y*. However, any ESS is a symmetric Nash equilibrium in a game between two fitness-maximizers [9].

9 This observation follows from the upper hemi-continuity that is implied by the topological properties stated in the result (see the discussion following result 3.13).
